# Research Progress on Ti_3_C_2_T_x_-Based Composite Materials in Antibacterial Field

**DOI:** 10.3390/molecules29122902

**Published:** 2024-06-18

**Authors:** Huangqin Chen, Yilun Wang, Xuguang Chen, Zihan Wang, Yue Wu, Qiongqiao Dai, Wenjing Zhao, Tian Wei, Qingyuan Yang, Bin Huang, Yuesheng Li

**Affiliations:** 1 Department of Stomatology, School of Stomatology and Ophthalmology, Hubei University of Science and Technology, Xianning 437100, China; chenhuangqin79@163.com (H.C.);; 2 Department of Computer Science and Technology, China Three Gorges University, Yichang 443002, China; 3 Hubei Key Laboratory of Radiation Chemistry and Functional Materials, Non-Power Nuclear Technology Collaborative Innovation Center, Hubei University of Science and Technology, Xianning 437100, China

**Keywords:** Ti_3_C_2_T_x_, antibacterial, classification, fabrication, application

## Abstract

The integration of two-dimensional Ti_3_C_2_T_x_ nanosheets and other materials offers broader application options in the antibacterial field. Ti_3_C_2_T_x_-based composites demonstrate synergistic physical, chemical, and photodynamic antibacterial activity. In this review, we aim to explore the potential of Ti_3_C_2_T_x_-based composites in the fabrication of an antibiotic-free antibacterial agent with a focus on their systematic classification, manufacturing technology, and application potential. We investigate various components of Ti_3_C_2_T_x_-based composites, such as metals, metal oxides, metal sulfides, organic frameworks, photosensitizers, etc. We also summarize the fabrication techniques used for preparing Ti_3_C_2_T_x_-based composites, including solution mixing, chemical synthesis, layer-by-layer self-assembly, electrostatic assembly, and three-dimensional (3D) printing. The most recent developments in antibacterial application are also thoroughly discussed, with special attention to the medical, water treatment, food preservation, flexible textile, and industrial sectors. Ultimately, the future directions and opportunities are delineated, underscoring the focus of further research, such as elucidating microscopic mechanisms, achieving a balance between biocompatibility and antibacterial efficiency, and investigating effective, eco-friendly synthesis techniques combined with intelligent technology. A survey of the literature provides a comprehensive overview of the state-of-the-art developments in Ti_3_C_2_T_x_-based composites and their potential applications in various fields. This comprehensive review covers the variety, preparation methods, and applications of Ti_3_C_2_T_x_-based composites, drawing upon a total of 171 English-language references. Notably, 155 of these references are from the past five years, indicating significant recent progress and interest in this research area.

## 1. Introduction

Bacterial infections pose a significant global threat, resulting in millions of fatalities annually and imposing severe consequences on both individuals and societies worldwide [1,2]. Traditional antimicrobial agents, primarily derived from chemically modified natural compounds known as antibiotics, have historically served as our primary defense against bacterial infections [3]. However, despite the development of numerous classes of antibiotics, the widespread and prolonged use of these agents has led to the emergence and spread of drug-resistant strains, escalating the peril posed by bacterial infections to a critical level and posing a profound threat to global public health [4,5,6]. Examples of such threats include wound infections [7], disease dissemination [8], as well as water and soil contamination [9,10]. In response to this urgent need, there is an imperative to develop novel and effective antimicrobial materials.

In recent decades, researchers have tirelessly explored and uncovered a myriad of antimicrobial agents, spanning a wide spectrum from organic to inorganic materials, and even nano-scale materials [11]. Notably, environmentally friendly nano-scale antimicrobial materials with low toxicity and high safety standards have emerged as a pivotal frontier, offering remarkable antimicrobial properties and witnessing burgeoning utilization across various antimicrobial applications [12]. These materials furnish enduring antimicrobial activity and critically thwart the emergence of drug-resistant strains, heralding a revolutionary breakthrough in the realm of antimicrobial intervention [13]. Common nanomaterials with antimicrobial properties include graphene, carbon-based materials, transition metal dichalcogenides, black phosphorus, noble metal nanoparticles, metal–organic frameworks (MOFs), conjugated polymers, and MXenes [14,15,16]. Among these, MXenes stand out owing to their superior surface area, metallic conductivity, light absorption capabilities, photothermal conversion efficiency, reactive oxide species (ROS) generation, and lower cellular toxicity, rendering them exceptional candidates as nano-scale antimicrobial agents [17,18,19,20].

MXenes, first synthesized in 2011, represent a novel class of two-dimensional layered ternary carbides prepared by Naguib et al. [21]. Their chemical formula is M_n+1_X_n_T_x_, where M refers to transition metals (Sc, Ti, V, Zr, Hf, Mo, Ta, and Nb), and X typically represents nitrogen (N) or carbon (C). “T” denotes surface terminations (e.g., -F, -Cl, -OH, and =O), and “x” (in T_x_) indicates the number of surface terminations, defining three common structures (M_2_XT_x_, M_3_X_2_T_x_, and M_4_X_3_T_x_) [22]. In the medical field, Ti_3_C_2_T_x_ and Ti_2_CT_x_ are the most widely utilized MXenes [23]. Ti_3_C_2_T_x_, derived from hydrofluoric acid (HF) etching of the Al layers from the Ti_3_AlC_2_ MAX phase [24], exhibits lower cytotoxicity, a higher specific surface area, and remarkable optical properties. Its photodynamic and photothermal sterilization effects under white and near-infrared (NIR) light hold immense promise in the field of antimicrobials [25,26,27]. However, as a single semiconductor material, Ti_3_C_2_T_x_ still presents drawbacks, particularly in photocatalytic applications, due to its poor solar response and carrier separation capabilities [28].

**Figure 1:**
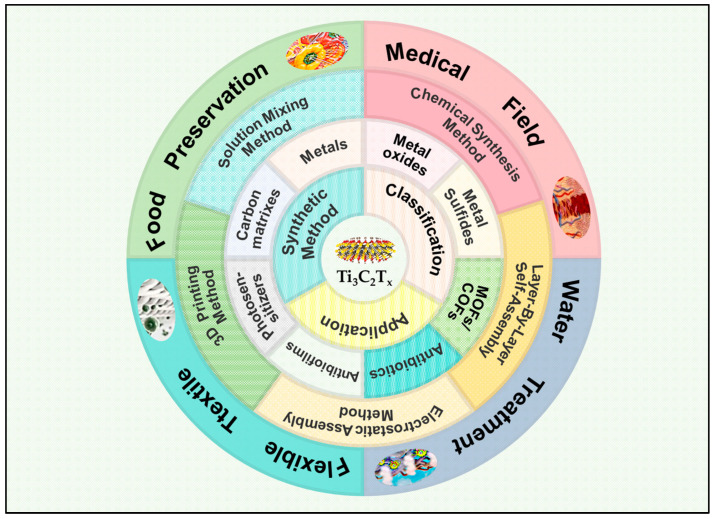
General applications of Ti_3_C_2_T_x_ composites in antibacterial field.

In recent years, researchers have developed numerous Ti_3_C_2_T_x_ composites to enhance antimicrobial performance. Combining Ti_3_C_2_T_x_ with noble metal nanoparticles, metal oxides, MOFs, and other materials significantly enhances its bactericidal capability. While mainstream reviews predominantly summarize the synthesis of Ti_3_C_2_T_x_ and its application in biomedical research, water purification, and biosafety research [29,30,31,32,33], comprehensive reviews on the classification and fabrication of Ti_3_C_2_T_x_ antimicrobial composites as well as their applications in antimicrobial fields remain relatively scarce [34]. Thus, this review explores the systematic classification of Ti_3_C_2_T_x_-based antimicrobial composites and their antibacterial efficiency, followed by a discussion of the main synthesis methods. Subsequently, we comprehensively review the latest advancements of Ti_3_C_2_T_x_-based composites in antimicrobial applications across the medical, water treatment, flexible fabric, food preservation, and industrial sectors. Finally, we outline the challenges and prospects, emphasizing the need for further research to elucidate microscopic mechanisms of action, achieve a balance between biocompatibility and antibacterial efficiency, and explore efficient, environmental friendly synthesis methods combined with smart technologies. The primary objective of this review is to elucidate the types of materials, the preparation method used for Ti_3_C_2_T_x_-based antibacterial materials, as well as their potential applications in the antibacterial field. In these domains, adjusting the shape and dimension of the final material, reducing the material cost, and enhancing industrial production efficiency will create the possibility of transformative applications (Figure 1).

## 2. Classification of the Ti_3_C_2_T_x_ Composite Antimicrobial Materials

With its outstanding physical and chemical properties, Ti_3_C_2_T_x_ holds immense promise to evolve into an even more superior and stable composite material. Currently, the main types of Ti_3_C_2_T_x_ composite antimicrobial materials encompass Ti_3_C_2_T_x_/metal composites, Ti_3_C_2_T_x_/metal oxide composites, Ti_3_C_2_T_x_/metal sulfide composites, Ti_3_C_2_T_x_/organic framework composites, Ti_3_C_2_T_x_/antibiotic composites, Ti_3_C_2_T_x_/antibiofilm agent composites, and Ti_3_C_2_T_x_/photosensitizer composites. Combining Ti_3_C_2_T_x_ with various types of antimicrobial materials enhances its antimicrobial properties through avenues such as photodynamic effects, photothermal effects, and physical cutting actions (Table 1).

**Table 1:** Classification of Ti_3_C_2_T_x_ composite antimicrobial materials.

Classification	Constituent	Cytocompatibility	Sterilization	Application	References
Metal	Au	The bacteria with materials in the dark showed essentially normal bacterial morphology	It effectively kills *Staphylococcus aureus* and prevents the development of biofilm.	Wound antibacterial healing auxiliary material	[35]
	Au	Not mentioned	The antibacterial efficiency is as high as 99.9%.	Multifunctional flexible pressure sensor	[36]
Metal oxide	V_2_O_5_	Not mentioned	It is an effective antibacterial agent against Gram-positive bacteria and Gram-negative bacteria.	Waste disposal	[37]
Metal sulfide	CuS	Not mentioned	It has good antibacterial activity against *Escherichia coli* and *Staphylococcus aureus*, with sterilization rates of 99.6% and 99.1%, respectively.	Disinfecting and antibacterial materials	[38]
Organic framework	MOFs	In the cytotoxicity test, the L929 cell survival rates were all above 75% after 12 h of incubation	After two rounds of bacterial inactivation and six months of indoor environment preservation, 99.9999% of Gram-negative *Escherichia coli* and Gram-positive *Staphylococcus aureus* can be eliminated.	Antibacterial material	[39]
Antibiotic	DOX	Not mentioned	It has an obvious inhibitory effect on Gram-negative *Escherichia coli* and Gram-positive *Bacillus subtilis* within 5 h.	Drug delivery	[40]
Antibiofilm	PVA	The relative viability changes of L929 cells after the co-incubation of the PVA hydrogel, Ag@M-PVA hydrogel, and Ag@M-H-PVA hydrogel extracts with L929 cells for 1 d and 3 d were above 85%	The inhibition rate of *Escherichia coli* under 808 nm near infrared radiation increased from 51.78% to 97.5%.	Tissue engineering repair material	[41]
Carbon matrix	Graphene	Only pure functionalized graphene shows some noteworthy cytotoxic activity after 24 h at a high concentration of 200 μg mL^−1^; however, after 48 h, the cytotoxicity of functionalized graphene and 50% Ti_3_C_2_T_x_—50% FG and 25% Ti_3_C_2_T_x_—75% FG increase to 80%, 55%, and 60%, respectively	For *Escherichia coli*, at the dosage of 200 mg/mL, Ti_3_C_2_ functionalized graphene nanocomposites can completely kill bacteria.	Biomedical application	[42]
	GO-PEI	Not mentioned	GO-PEI/MXene folded microspheres have strong adsorption capacity, and the antibacterial effect can prolong the service life.	Molecular imprinting technique	[43]
Natural antibacterial material	Chitosan	Not mentioned	The sterilization effect is close to 100%.	Health monitoring	[44]

### 2.1. Single Ti_3_C_2_T_x_

As a quintessential MXene material, Ti_3_C_2_T_x_ plays a pivotal role in various domains, particularly showcasing significant breakthroughs in antibacterial applications in recent years [45]. Its outstanding antibacterial efficacy primarily arises from two mechanisms: physical slicing and photocatalytic effects [46]. Moreover, the method of preparation exerts a substantial influence on its antibacterial performance. Compared to the conventional wet chemical etching method, which lacks mechanical force and yields Ti_3_C_2_T_x_ lacking sharp edges, Ti_3_C_2_T_x_ prepared via the one-step mechanical exfoliation method exhibits superior “nanoscale knife effects”. Its irregular sharp edges can disrupt bacterial cell walls, leading to enhanced bacteriostatic effects [47].

Two-dimensional Ti_3_C_2_T_x_ nanosheets, utilizing the strategy of tetramethylammonium hydroxide-driven intercalation and delamination, demonstrate excellent biocompatibility and synergistic photothermal–photodynamic antibacterial activity against *Escherichia coli* (*E. coli*) and *Staphylococcus aureus* (*S. aureus*). Compared to traditionally HF-exfoliated Ti_3_C_2_T_x_ nanosheets, photothermal–photodynamic Ti_3_C_2_T_x_ nanosheets exhibit significant antibacterial enhancement at concentrations of 0.5 mg/mL or 1.0 mg/mL, achieving nearly 100% bacterial inhibition due to improved biofilm disruption and leakage of bacterial contents under NIR illumination [48]. Furthermore, certain photothermal treatments applied to Ti_3_C_2_T_x_ bolster its antibacterial performance. For instance, photoactivated Ti_3_C_2_T_x_ demonstrates robust thermal effects and potent antibacterial activity, and it is capable of suppressing the expression of inflammatory factors and combating methicillin-resistant *Staphylococcus aureus* (MRSA) infections [49].

### 2.2. Ti_3_C_2_T_x_/Metal Composites

Metal nanoparticles exhibit unique surface plasmon resonance effects, excellent conductivity, and heat resistance. When doped with semiconductor materials to form composites, they can significantly enhance photothermal efficiency. Upon binding with Ti_3_C_2_T_x_, metal nanoparticles infiltrate the interlayer gaps, markedly enhancing the antibacterial activity of the composite. Common Ti_3_C_2_T_x_/metal composites include Cu/Ti_3_C_2_T_x_, Au/Ti_3_C_2_T_x_, Ag/Ti_3_C_2_T_x_, and Pt/Ti_3_C_2_T_x_ [50,51,52,53].

**Figure 2:**
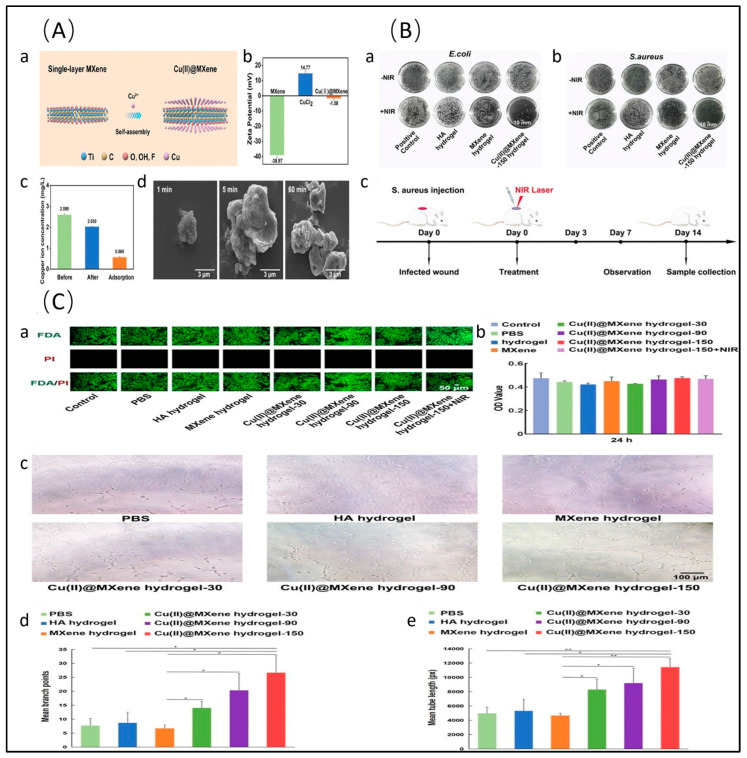
Ti_3_C_2_T_x_/metal composites [59]. (**A**) Synthesis and characterization of Cu(II)@MXene complex. (**a**) Schematic diagram of the synthesis process of Cu(II)@MXene complex. (**b**) Zeta potential of MXene, CuCl2, and Cu(II)@MXene. (**c**) Concentration of Cu^2+^ in the supernatant before and after electrostatic self-assemble between Cu^2+^ and MXene. (**d**) The SEM images of Cu(II)@MXene at different time points. (**B**) Antibacterial properties of Cu(II)@MXene hydrogel and promotion of infected wound healing in vivo. Total view of bacterial colonies formed by *E. coli* (**a**) and *S. aureus* (**b**) after different hydrogel treatments. (**c**) Schematic diagram of the construction of mouse skin infection model. (**C**) In vitro cytocom-patibility and angiogenesis property of Cu(II)@MXene hydrogel. (**a**) Live/dead staining images of the L929 cells after being treated with different materials for 24 h. (**b**) CCK-8 results of the L929 cells after treatment with different materials for 24 h. (**c**) Tube formation assay of HUVECs treated by different materials. The quantitative analysis of formed (**d**) branch points and (**e**) tube length, respectively. Statistic results: * *p* < 0.05. ** *p* < 0.01.

Au/Ti_3_C_2_T_x_ photothermal films can completely deactivate *E. coli* within 20 min, demonstrating remarkable photothermal antibacterial performance [54]. Ti_3_C_2_T_x_-Au nanoparticles kill over 99% of bacteria with a low dose of nanoparticle suspension (50 μg/mL) under visible light irradiation for just 10 min [55]. Ti_3_C_2_T_x_-Au nanobipyramids demonstrate broad-spectrum antibacterial properties for various bacterial strains (bactericidal rates all above 95%) within 8 min under 808 nm laser irradiation with a photothermal conversion efficiency of 50.41% [56]. Two-dimensional core–shell Ti_3_C_2_T_x_@Au nanocomposites expand the infrared absorption range from NIR-I (650–950 nm) to NIR-II (1000–1350 nm) [57]. Incorporating Cu and Ag into the interlayer spacing of Ti_3_C_2_T_x_ also enhances antibacterial activity [50,58]. Based on electrostatic self-assembly between Cu^2+^ and MXene, Cu(II)@Ti_3_C_2_T_x_ photothermal composites embedded in hyaluronic acid hydrogels are formulated as antibacterial dressings, offering easy application to wounds of diverse shapes and ensuring long-term wound protection. Additionally, these readily prepared Cu(II)@Ti_3_C_2_T_x_ composites simultaneously function as a photothermal antibacterial barrier, ROS scavenger, and angiogenesis promoter, thereby expediting the healing process of infected wounds [59] (Figure 2). The anchoring of Ag nanoparticles on Ti_3_C_2_T_x_ and covalent organic framework (COF) composites results in exceptional antibacterial properties against *S. aureus* and *Pseudomonas aeruginosa* (*P.aeruginosa*). The synergistic catalytic effect of Ag and Ti_3_C_2_T_x_ significantly reduces the work function along the interface, while the built-in electric field between the layers drives rapid carrier migration, thereby effectively improving catalytic performance [60]. In addition to binding with individual metal particles, the combination of Ti_3_C_2_T_x_ with bimetallic nanoparticles significantly enhances antibacterial efficiency. AgPd or AgAu bimetallic nanocrystals, sandwiched between a Ti_3_C_2_T_x_ nanosheet and polydopamine (PDA) layer, possess a cage-like nanostructure, strong absorption in the NIR region, and a local surface plasmonic resonance effect. These characteristics are conducive to high catalytic efficiency and antibacterial performance. Moreover, owing to the presence of Ag ions and photothermal coupling antibacterial properties, composite nanosheets exhibit effective antibacterial activity against both Gram-negative (*E. coli*) and Gram-positive (*S. aureus*) bacteria [61,62].

### 2.3. Ti_3_C_2_T_x_/Metal Oxide Composites

Besides its interaction with metal ions, Ti_3_C_2_T_x_ enhances its antibacterial efficacy through combining with metal oxides. The antibacterial performance of metal oxides is closely related to their ability to generate oxygen free radicals and their physical interaction with bacterial membranes. Facilitated by their porous structure and numerous channels, metal oxides have high-density, evenly distributed catalytic active centers, which promote the thorough penetration of small-molecule substrates, optimizing the transport and diffusion of products to achieve antibacterial effects [63]. Ti_3_C_2_T_x_ nanosheets, with their large surface area and excellent hydrophilic properties, are considered ideal carriers for metal oxides [64]. Common Ti_3_C_2_T_x_/metal oxide composite materials include TiO_2_/Ti_3_C_2_T_x_, Cu_2_O/Ti_3_C_2_T_x_, ZnO/Ti_3_C_2_T_x_, and various ferrite/Ti_3_C_2_T_x_ composites [65,66,67,68]. Among these, TiO_2_/Ti_3_C_2_T_x_ composites have been extensively studied [69].

Two-dimensional membranes constructed from in situ-generated nano TiO_2_/Ti_3_C_2_T_x_ layers exhibit excellent photocatalytic performance. Compared to non-photocatalytic Ti_3_C_2_T_x_ membranes, TiO_2_/Ti_3_C_2_T_x_ membranes under ultraviolet irradiation demonstrate a very high flux recovery ratio (80%) and over 95% resistance to *E. coli* [70]. The presence of nano TiO_2_ particles as interlayer support layers widens the interlayer channels, enhances membrane permeability, and improves self-cleaning properties and long-term membrane operational stability. Combining the antibacterial properties of copper, a multifunctional Cu_2_O@Ti_3_C_2_T_x_/α-tricalcium phosphate scaffold with internal and external sandwiching constructed via 3D printing utilizes the excellent photothermal properties of Ti_3_C_2_T_x_ to control programmed temperature. This includes brief PTT to rapidly eliminate early bacteria and periodic low photothermal stimulation to promote bone tissue growth [71]. Simultaneously, the slow release of endogenous copper ions strengthens antibacterial efficiency, promotes angiogenesis, and improves the repair effect. ZnO/Ti_3_C_2_T_x_ heterojunctions synthesized by the hydrothermal growth of ZnO on the surface of lamellar Ti_3_C_2_T_x_ exhibit superior photothermal performance compared to ZnO and Ti_3_C_2_T_x_ alone [72]. Local thermal effects not only disrupt the bacterial membrane integrity but also promote the release of Zn^2+^ ions, further enhancing antibacterial performance with a sterilization rate of 100% at a 150 μg·mL(-1) concentration (superior to either ZnO or Ti_3_C_2_T_x_). CuFe_2_O_4_-Ti_3_C_2_T_x_ antibacterial heterojunctions, constructed by in situ growth, produce high heat and exogenous ROS under NIR light irradiation [73]. Additionally, they consume glutathione in bacteria through Fenton-like reactions and generate endogenous ROS, resulting in a robust and lasting antibacterial cascade effect. Furthermore, novel magnetic Ti_3_C_2_T_x_@Fe_3_O_4_/Au/PDA nanosheets exhibit excellent photothermal-magnetic decoupling antibacterial performance [74]. The antibacterial effect against *E. coli* and *S. aureus* at a concentration of 120 μg/mL approaches 100%.

TiO_2_/Ti_3_C_2_T_x_ membranes excel in photocatalytic performance and water flux recovery for water treatment applications, while the Cu_2_O@Ti_3_C_2_T_x_/α-tricalcium phosphate scaffold leverages its multifunctional properties to promote bone tissue growth and enhance antibacterial efficiency for medical implants. ZnO/Ti_3_C_2_T_x_ heterojunctions demonstrate superior photothermal performance and antibacterial action through the combined effects of local thermal effects and Zn^2+^ ion release. CuFe_2_O_4_-Ti_3_C_2_T_x_ antibacterial heterojunctions, on the other hand, produce high heat and exogenous ROS under NIR irradiation, offering another effective antibacterial solution. Each material exhibits unique advantages tailored for specific applications, whether it be water treatment, medical implants, or antibacterial applications.

### 2.4. Ti_3_C_2_T_x_/Metal Sulfide Composites

Given the escalating issue of antibiotic resistance, environmentally friendly photoelectric materials hold promise as alternatives to antibiotics. Metal sulfides, characterized by excellent biocompatibility, a low cost, and particularly noteworthy photothermal properties, have garnered special attention. They possess economic viability, chemical stability, and the ability to easily couple with other semiconductors [75]. For instance, the interface Schottky heterojunction designed by the contact potential difference between Bi_2_S_3_ and Ti_3_C_2_T_x_ inhibits electron backflow and boosts charge transfer and separation, thereby intensively improving the amount of ROS under 808 nm NIR radiation [76]. This enables the material to achieve a bactericidal rate of 99.86% of *S. aureus* and 99.92% of *E. coli* with the assistance of hyperthermia within 10 min. BiOI@Bi_2_S_3_/Ti_3_C_2_T_x_ composite materials synthesized through the in situ vulcanization of two-dimensional Ti_3_C_2_T_x_ with hollow flower-shaped BiOI achieve a photothermal conversion efficiency of 57.8% under 808 nm laser irradiation, thereby boosting PTT antibacterial effects [77]. Meanwhile, the close contact of heterojunction interfaces enhances the charge transfer and suppresses electron–hole recombination, improving the antibacterial performance of PDT. The combined PTT and PDT antibacterial actions result in efficiencies of 99.7% and 99.8% against *P. aeruginosa* and *S. aureus*, respectively. Ti_3_C_2_T_x_/ZnIn_2_S_4_ heterojunctions achieve controllable electromagnetic properties through the dual optimization of intercalated nano interfaces of Ti_3_C_2_T_x_ and an S vacancy structure design, exhibiting strong antibacterial activity [78]. MoS_2_ demonstrates excellent photocatalytic performance and infrared photothermal effects, making it suitable for various antibacterial pathways such as photocatalytic antibacterial, enzyme-like catalytic antibacterial, physical antibacterial, and photothermal-assisted antibacterial pathways [79]. For example, Ti_3_C_2_T_x_/MoS_2_ heterojunctions induce local heating and increase extracellular ROS levels, resulting in bacterial inactivation under NIR laser irradiation [80]. Meanwhile, Mo^4+^ ions readily invade ruptured bacterial cell membranes, inducing intracellular ROS and depleting intracellular glutathione. Under the pressure of “ROS hurricanes” from both internal and external sides, bacteria are hugely slaughtered. When MoS_2_ grows in a vertical arrangement on Ti_3_C_2_T_x_, it damages the peptidoglycan mesh in the bacterial wall, leading to significant morphological changes and thus bacteriostatic effects [81]. In addition, CuS nanoparticles also can rapidly convert light energy into heat under NIR light irradiation, making them suitable for photothermal sterilization [82]. Accordion-like Ti_3_C_2_T_x_@CuS structures enhance the separation efficiency of CuS electron–hole pairs, generating more ROS to kill bacteria [83]. Moreover, with the synergistic effect of sustained copper ion release, they maintain over 90% long-term sterilization ability within 30 days. Furthermore, FeS@lauramidopropyl betaine@Ti_3_C_2_T_x_ nanoenzymes exhibit catalytic activity similar to peroxidases, promoting the production of hydroxyl radicals by catalyzing the decomposition of H_2_O_2_ [84]. They demonstrate excellent antibacterial activity against typical Gram-negative *E. coli* and Gram-positive *S. aureus*, integrating chemical kinetics and PTT to provide an effective strategy for bacterial inhibition and wound healing.

### 2.5. Ti_3_C_2_T_x_/Organic Frameworks Composites

Organic frameworks refer to multidimensional network structures composed of organic molecules or polymers. These materials exhibit highly ordered microstructures and offer a high degree of tunability in terms of performance. Currently, in the field of antibacterial materials, the main organic frameworks that are being combined with Ti_3_C_2_T_x_ are MOFs and COFs.

**Figure 3:**
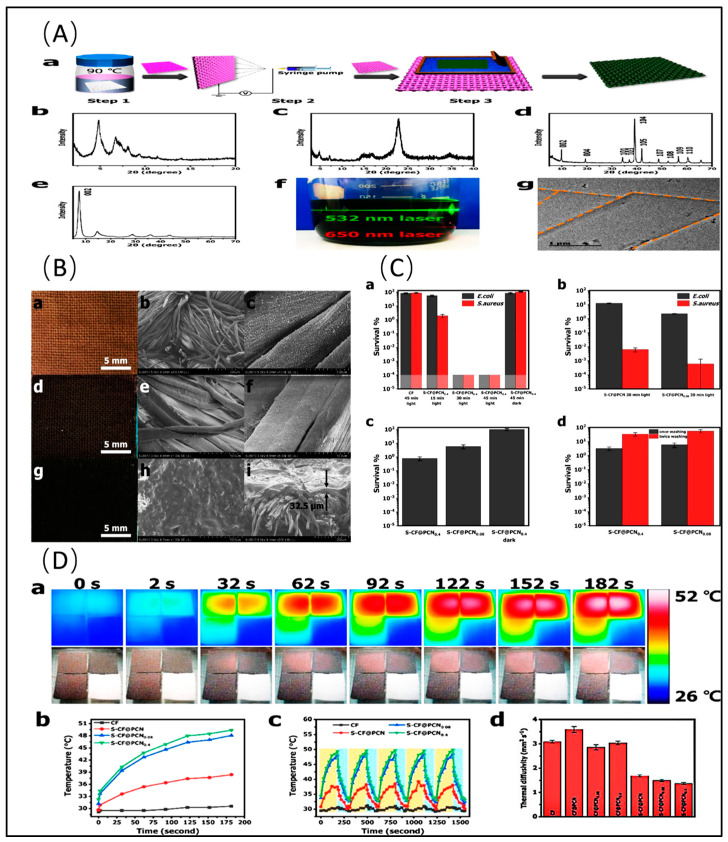
Ti_3_C_2_T_x_/organic frameworks [87]. (**A**,**B**) Design and characterization of S-CF@PCNx. (**A**) (**a**) Schematic illustration of the production of S-CF@PCNx. (**b**–**e**) XRD characterization of PCN-224, CF@PCN, Ti_3_AlC_2_ MAX, and Ti_3_C_2_ MXene. (**f**) Digital photograph showing the Tyndall scattering effect of the Ti_3_C_2_ MXene nanosheet suspension. (**g**) TEM image of Ti_3_C_2_ MXene nanosheets. (**B**) (**a**–**c**) Digital photograph and SEM images of CF@PCN. (**d**–**f**) Digital photograph and SEM images of CF@PCN0.4. (**g**) Digital photograph of S-CF@PCN0.4. (**h**–**i**) Surface and cross section observation of S-CF@PCN0.4. (**C**) Photodynamic antibacterial properties. (**a**,**b**) Antibacterial photodynamic inactivation study against *E. coli* and *S. aureus*. (**c**) Reusability of antibacterial activity of the materials against E. coli under 30 min illumination. (**d**) Antibacterial activity against *E. coli* after one and two washes under 30 min illumination. The light source for bacterial inactivation was provided by a Xe lamp (500 W, 15 cm sample distance, λ ≥ 420 nm). (**D**) Photothermal and photothermochromic effect. (**a**) Surface thermal and digital images under Xe lamp illumination (500 W, 15 cm sample distance, λ ≥ 420 nm). Left top: S-CF@PCN0.08; right top: S-CF@PCN0.4; left bottom: S-CF@PCN; and right bottom: CF. (**b**) Temperature rise curves of CF, S-CF@PCN, S-CF@PCN0.08, and S-CF@PCN0.4. (**c**) Photothermal heating curves of CF, S-CF@PCN, S-CF@PCN0.08, and S-CF@PCN0.4 for five cycles. (**d**) Thermal diffusivity of all of the prepared fabrics.

MOFs are a class of emerging hybrid porous materials composed of clusters of metal ions or clusters connected by organic linkers. They possess advantages such as a high surface area, a tunable structure, chemical functionality, and host–guest interactions, making them widely applicable in various fields [85]. MOFs have attracted considerable attention regarding antibacterial potential as they select diverse metal ions and organic ligands to control their outstanding physical and chemical properties. For instance, utilizing Ti_3_C_2_T_x_ as a conductive carrier and UiO66-NH_2_ (C_48_H_30_N_6_O_32_Zr_6_) as positively charged nano-MOFs, Ti_3_C_2_T_x_-based Schottky heterojunctions exhibit inhibition rates greater than 95% against *E. coli* and *S. aureus* under visible light irradiation [86]. During the formation of the Schottky heterojunction, UiO66-NH_2_ nanoparticles grow in situ on the surface and inner surface of Ti_3_C_2_T_x_, not only combining the antibacterial properties of Ti_3_C_2_T_x_ but also effectively reducing its bandgap energy, thereby enhancing its photocatalytic activity. Smart photothermochromic self-disinfecting textiles prepared through the in situ growth of porphyrin-type MOF (PCN-224, C_144_H_112_N_12_O_64_Zr_15_) and the electrospray coating of Ti_3_C_2_T_x_ colloid inactivate 99.9999% of *E. coli* and *S. aureus* within 30 min. Loading nano-silver on the basis of PCN-224/Ti_3_C_2_T_x_ not only synergistically enhances the antibacterial effect through photocatalytic single-state oxygen generated by PCN-224 and the heat generated by Ti_3_C_2_T_x_, but also promotes the degradation of nano-silver to release silver ions, achieving rapid bacterial inactivation and persistent bacterial inhibition [87] (Figure 3).

COFs are porous crystalline materials formed by covalently connecting organic molecules. They possess advantages such as tunable bandgaps and large surface areas. However, compared to traditional photocatalytic bactericides, the antibacterial effect of COFs is not ideal, mainly due to the low separation efficiency of electron–hole pairs. An effective solution is to modify the surface of COFs to enhance the separation efficiency and migration rate of photo-generated charge carriers [88]. The in situ growth of TpPa-1-COF (C_15_H_14_N_2_O_6_) on Ti_3_C_2_T_x_ through Schiff base reaction improves the antibacterial rates to 98.90% and 99.62%, respectively, against *S. aureus* and *P. aeruginosa*, which are 50% and 33% higher than those of pure TpPa-1-COF [89]. Electrochemical impedance spectroscopy and photoluminescence spectra indicate that the migration rate of photo-generated carriers between layers is increased, thereby achieving efficient photocatalysis and reducing photocorrosion.

### 2.6. Ti_3_C_2_T_x_/Antibiotic Composites

Antibiotics, primarily secondary metabolites produced by bacteria, fungi, or other microorganisms, or synthetic analogs thereof, exert their bactericidal effect by targeting specific processes in bacterial cell DNA, RNA, and protein synthesis, thereby disrupting normal bacterial metabolism and inhibiting bacterial growth [90]. However, the overuse of antibiotics can lead to the emergence of antibiotic-resistant strains [91,92].

**Figure 4:**
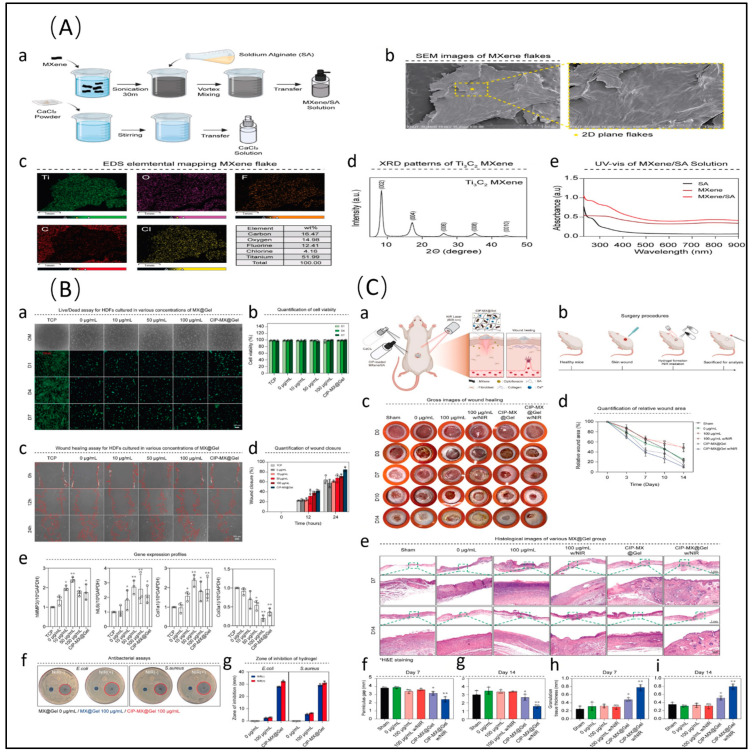
Ti_3_C_2_T_x_/antibiotic composite materials [94]. (**A**) Preparation and characterization of the MXene/SA hydrogel (MX@Gel). (**a**) Temperature change of MX@Gel containing MXene at various concentrations (0, 10, 50, 100 μg/mL) under near-infrared (NIR) laser irradiation (808 nm, 0.5 W/cm^2^). (**b**) Heating and cooling curves of MX@Gel by laser on/off cycle. (**c**) MX@Gel temperature change graph according to various laser densities (0.50, 1.00, 1.50, 2.00 W/cm^2^). (**d**) Schematic of loading ciprofloxacin on MXene surface. (**e**) UV–vis absorption spectrum of CIP-loaded MXene (CIP-MX). (**B**) Biocompatibility and cell migration of the CIP-MX@Gel with antibacterial property. (**a**) Optical microscopy and Live/dead staining images of human dermal fibroblasts cultured on MX@Gel at 1, 4, and 7 days. (**b**) quantification of cell viability of MX@Gels. (**c**) Wound healing scratch assay of MX@Gel at different concentrations. (**d**) Quantification of cell migration (n = 3). (**e**) Gene expression profiles of MX@Gels (n = 3). (**f**) Antibacterial gross images of *E. coli* and *S. aureus* in CIP-MX@Gel using smear plate method. (**g**) Quantification of the zone of inhibition of CIP-MX@Gel (n = 3). All data represent mean ± SD. * *p* < 0.05, ** *p* < 0.01. The symbol * indicates comparisons with 0 μg/mL. Scale bars = 100 μm (**a**,**c**). (**C**) In vivo wound healing effect of CIP-MX@Gel in mice. (**a**) Schematic diagram of the formation process of sprayable CIP-MX@Gel, and drug release by NIR irradiation (**b**) Schematic diagram of the experimental healing process in vivo. (**c**) Wound images at days 0, 3, 7, 10 and 14 (Silicone isolator diameter = 9 mm). (**d**) quantification of relative wound area. (n ≥ 3) (**e**) Hematoxylin and eosin (H&E) staining images on day 7 and 14 of wound tissues treated in different groups. (**f**,**g**) Quantification of the panniculus gap at day 7 and day 14 in wound tissue treated with different groups. (**h**,**i**) Quantification of the granulation tissue thickness at day 7 and day 14 in wound tissue treated with different groups. All data represent mean ± SD. * *p* < 0.05, ** *p* < 0.01. The symbol * indicates comparisons with Sham. Scale bars = 1 mm (**e**), 100 μm (magnified images, **e**).

Combining Ti_3_C_2_T_x_ with antibiotics not only enhances antibacterial efficiency, but also effectively reduces the use of antibiotics. Common antibiotics, such as amoxicillin, ciprofloxacin, and tobramycin, are often employed in such composite materials. For instance, electrospun nanofiber membranes produced by loading amoxicillin onto a Ti_3_C_2_T_x_ and polyvinyl alcohol mixture achieve a killing efficiency rate of 96.1% against *S. aureus* upon laser irradiation, while the effective bactericidal rate is 99.1% when combined with an NIR laser [93]. These composite nanofiber membranes not only control the release of amoxicillin to combat bacterial infections, but also utilize Ti_3_C_2_T_x_ to convert NIR laser into heat, thereby destroying the non-cellular components of bacteria and synergistically leading to bacterial inactivation. Similarly, the combination of ciprofloxacin and Ti_3_C_2_T_x_ into nanocomposite materials achieve an outstanding in vitro bactericidal effect of >99.99999% against MRSA [45]. This composite material demonstrates long-term inhibition in a mouse MRSA-induced abscess model, avoiding bacterial rebound post PTT. Ciprofloxacin-Ti_3_C_2_T_x_ nanocomposite materials can be prepared as sprayable hydrogels, offering excellent photothermal conversion capability and biocompatibility, ultimately enhancing antibacterial activity [94] (Figure 4). Multifunctional implants composed of tobramycin and Ti_3_C_2_T_x_ nanosheets exhibit robust antibacterial properties against both Gram-negative and Gram-positive bacteria [95]. Importantly, these implants demonstrated excellent cellular compatibility and osteogenic potential, promoting bone cell proliferation, diffusion, alkaline phosphatase activity, calcium matrix mineralization, and in vivo bone integration. In addition, encapsulating tick-derived antimicrobial peptides as model drugs in hydrogels with Ti_3_C_2_T_x_ nanoparticles resulted in exceptional antibacterial properties, excellent biocompatibility, and superior skin tissue regeneration capabilities, offering broad applications in wound healing [96].

### 2.7. Ti_3_C_2_T_x_/Antibiofilm Composites

Antibiofilm agents refer to compounds and drugs that inhibit the formation or disrupt the structure of bacterial biofilms. Common antibiofilm agents include PDA, polyethyleneimine, polycaprolactone, and others.

**Figure 5:**
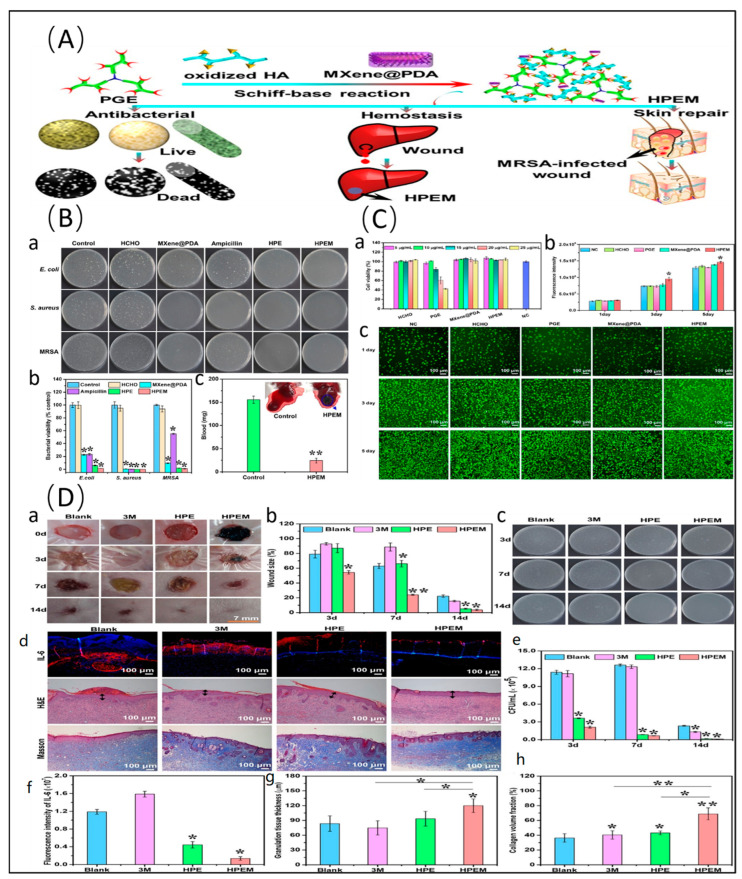
Ti_3_C_2_T_x_/antibiofilm composite [103]. (**A**) The fabrication and application of HPEM scaffolds in multidrug-resistant bacteria-infected wound healing. (**B**) Antibacterial activity and hemostatic ability assay. (**a**) Bacteria clones and (**b**) bacterial viability against E. coli, S. aureus, and MRSA treated with different samples. (**c**) Total blood loss and photographs from the damaged livers after 60 s of treatment with HPEM scaffolds and the control (* *p* < 0.05, ** *p* < 0.01, n = 3). (**C**) Cytotoxicity and cell proliferation evaluations of scaffolds. (**a**) Cell viability of HPEM scaffolds and each composite in L929 cells at different concentrations for 24 h. (**b**) LIVE/DEAD staining images of L929 cells at 1, 3, and 5 days after treated with HPEM scaffolds and controls (10 μg/mL) (live cells: green, dead cells: red, scale bar: 100 μm, n = 3). (**c**) Fluorescence intensity of L929 cells treatment with HPEM scaffolds and controls at 1, 3, and 5 days (* *p* < 0.05, n = 5). Cells without any treatment were used as a negative control (NC). (**D**) In vivo anti-infection and MRSA-infected wound healing. (**a**) Representative skin wound images at 0, 3, 7, and 14 days and (**b**) wound area size of the HPEM scaffolds and controls (* *p* < 0.05, ** *p* < 0.01). (**c**) Images of MRSA colonies growing on the agar plates derived from the homogenized infected tissues after various treatments at 3, 7, and 14 days. (**d**) Quantitative bacterial colonies densities based on (**c**). (**e**) IL-6 (red) immunofluorescence images at day 3, and H&E and Masson’s trichrome staining of wound tissues at day 14; the black arrows show the granulation layers in wound beds. Corresponding quantification of (**f**) fluorescence intensity of IL-6, (**g**) granulation tissue thickness, and (**h**) collagen content in wound tissues based on (**e**) (scale bar = 100 μm, n = 6; * *p* < 0.05 and ** *p* < 0.01).

PDA is renowned for its excellent photothermal properties. When PDA is combined with Ti_3_C_2_T_x_, its photothermal effects and free radical scavenging capabilities are enhanced, and it can even produce Fenton-like activity, exhibiting promising antibacterial and anti-inflammatory strategies [97,98]. Coating in situ-grown Ti_3_C_2_T_x_@gold nanorods with PDA results in synergistic photodynamic antibacterial activity [99]. Composite nanoparticles generate more ^1^O_2_ under 660 nm laser irradiation with the increase in the PDA content, reducing the minimum inhibitory concentration against *E. coli* and *S. aureus* to approximately 0.07 mg/mL. Additionally, the PDA coating further enhances the antioxidative and antibacterial abilities of Ti_3_C_2_T_x_, providing the scaffold with excellent ROS scavenging capacity [100] and tissue adhesion properties [101], thereby promoting wound healing in diabetic infection wounds [102] and wounds infected with multidrug-resistant bacteria [103] (Figure 5). Moreover, PDA coating can passivate the sharp edges of Ti_3_C_2_T_x_ nanosheets, preventing damage to normal tissue cells [104].

Polyethyleneimine and Ti_3_C_2_T_x_-modified sodium alginate aerogels also exhibit significantly reinforced mechanical strength, achieving a 99.99% antibacterial rate against *S. aureus* and *E. coli* within 2 h [105]. Polycaprolactone is commonly used to prepare electrospun nanofiber membranes with biocompatibility and biodegradability [106]. The presence of Ti_3_C_2_T_x_ helps reduce the diameter of electrospun nanofibers, significantly enhancing the hydrophilicity, electroactivity, and antibacterial activity of membranes [107]. Furthermore, this nanofiber membrane significantly promotes the adhesion, proliferation, and migration of NIH 3T3 cells under electrical stimulation, thereby accelerating wound closure, increasing granulation tissue formation, collagen deposition, and wound vascularization, making it a promising multifunctional wound dressing [108].

In summary, the combination of Ti_3_C_2_T_x_ with antibiofilm agents offers a versatile approach for enhancing antibacterial activity, promoting wound healing, and preventing biofilm formation.

### 2.8. Ti_3_C_2_T_x_/Photosensitizer Composites

**Figure 6:**
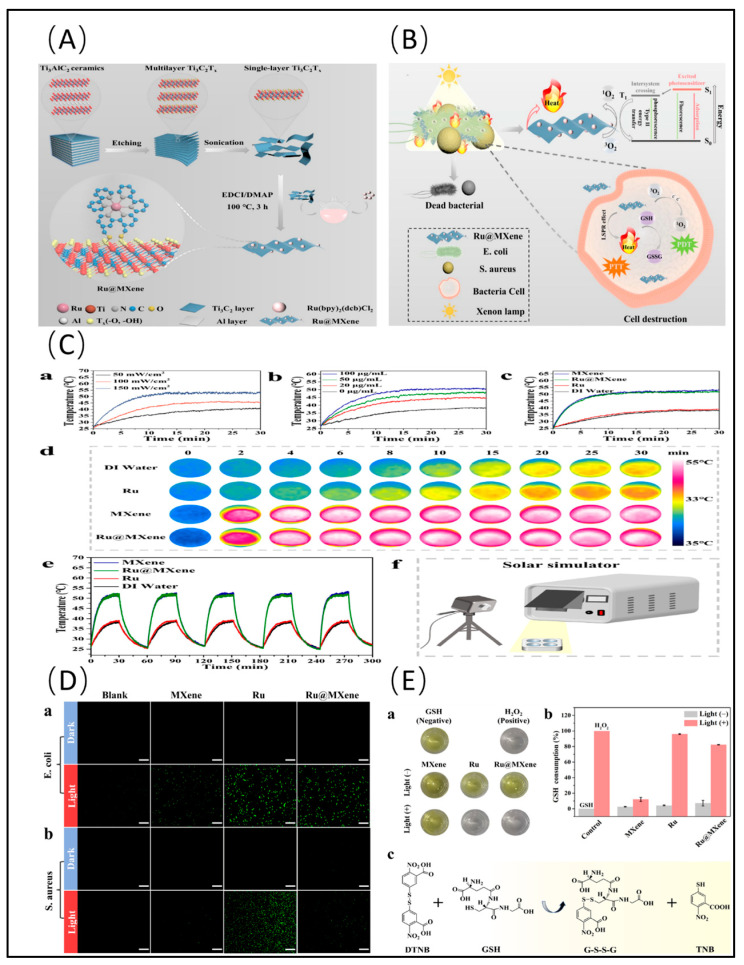
Ti_3_C_2_T_x_/photosensitizer composite material [110]. (**A**) A sSchematic illustration of the preparation of Ru@MXene nanocomposites. (**B**) A sSchematic diagram of Ru@MXene as a photoconverting agent to produce heat and 1O2 for achieving PTT/PDT antibacterial activity. (**C**) Photothermal property assessments: photothermal heating curve of Ru@MXene (**a**) with gradient concentrations; and (**b**) with different power density. (**c**) Photothermal heating curve of various samples with a power density of 150 mW/cm^2^. (**d**) Real-time infrared thermal images of different samples. (**e**) Photothermal periodic curve of Ru@MXene. (**f**) Schematic diagram of simulated sunlight irradiation samples. (**D**) Antibacterial mechanism evaluations involving intracellular ROS level detection.: The fFluorescence intensity of intracellular ROS induced by different samples with or without xenon lamp illumination (150 mW/cm^2^, 30 min) in (**a**) *E. coli* and (**b**) *S. aureus*. (Scale bar: 50 μm). (**E**) Antibacterial mechanism evaluations involving GSH consumption: (**a**) Photographs of the color change in GSH solution after incubating with various materials with or without xenon lamp illumination (150 mW/cm^2^, 30 min). (**b**) Corresponding GSH consumption rate (n = 3). (**c**) Chemical reaction between DTNB and GSH.

Photosensitizers are compounds capable of absorbing specific wavelengths of light energy and undergoing photodynamic reactions to produce ROS, which play a crucial role in PDT. Common photosensitizers include indocyanine green (ICG), ruthenium (Ru), and zinc phthalocyanine (ZnTCPP). Loading ICG onto Ti_3_C_2_T_x_ nanosheets combines the photothermal effect of Ti_3_C_2_T_x_ with the photodynamic effect of ICG, resulting in a 100% increase in the vitality loss of *S. aureus* under NIR light spectrum [109]. Ti_3_C_2_T_x_ nanocomposites based on Ru(II) complex grafting achieve almost 100% bactericidal activity against *E. coli* (200 μg/mL) and *S. aureus* (100 μg/mL) upon exposure to a xenon lamp [110] (Figure 6). ZnTCPP-modified Ti_3_C_2_T_x_ captures bacteria via surface electrostatic interactions [111]. The Schottky junction formed between Ti_3_C_2_T_x_ and ZnTCPP promotes visible light utilization, accelerates charge separation, enhances the migration rate of photogenerated charges, and ultimately improves photocatalytic activity. This visible light-responsive organic–inorganic hybrid achieves antibacterial efficiencies of 99.86% and 99.92% against *S. aureus* and *E. coli*, respectively, within 10 min. Porphyrin MOFs combined with Ti_3_C_2_T_x_ not only kill drug-resistant bacteria by generating abundant ROS but also promote the proliferation of stem cells by regulating the cell cycle, DNA replication, and apoptosis [112]. They promote osteogenic differentiation through key signaling pathways, including calcium, Wnt, and TGF-beta signaling pathways. A sandwich structure composed of TiO_2_, Ti_3_C_2_T_x_ and a photosensitizer (PANi (Ni-phytate)) achieves the bidirectional transfer of hot electrons under 808 nm NIR light irradiation, elevating the photothermal conversion efficiency to 43.3% and realizing a bactericidal effect of 99.9% against *E. coli* [113]. This study presents a novel approach to construct NIR light-induced antibacterial materials, realizing the synergistic effect of photocatalytic therapy and PTT.

With the increasing demand for antibacterial materials, Ti_3_C_2_T_x_-based antibacterial materials are rapidly evolving from singular components towards composite materials. This includes using Ti_3_C_2_T_x_ as a carrier to load various antibacterial drugs or directly incorporating it into 3D networks, such as antibacterial gels or films. Through this approach, the advantages of different antibacterial substances can be combined to achieve a broader antibacterial spectrum and more durable antibacterial effects. In recent years, utilizing the photocatalytic properties of Ti_3_C_2_T_x_ for photothermal treatment has emerged as a new trend in developing antibacterial effects, especially by combining Ti_3_C_2_T_x_ with other photocatalytic materials to enhance antibacterial performance through increased photocatalytic activity. This evolution demonstrates diversification strategies in the development of Ti_3_C_2_T_x_-based antibacterial materials, where wise material selection and combination offer new possibilities for developing efficient antibacterial materials.

## 3. Preparation Methods

The preparation methods for Ti_3_C_2_T_x_ antibacterial composite materials are diverse and can be broadly categorized into the following approaches: the solution mixing method, chemical synthesis method, layer-by-layer self-assembly method, electrostatic assembly method, and 3D printing. These methods offer various ways to combine Ti_3_C_2_T_x_ with other materials to enhance its antibacterial properties. The choice of preparation method significantly influences the structure and performance of Ti_3_C_2_T_x_ antibacterial composite materials (Table 2).

**Table 2:** Preparation methods of Ti_3_C_2_T_x_-based antibacterial composite materials.

Classification	Constituent	Sterilization	Application	Reference
Solution mixing	Cationic polymer poly-L-lysine, Ti_3_C_2_	At the concentration of 200 mg L^−1^, the number of living cells of *E. coli*. decreased by two orders of magnitude.	Biotechnology or nanomedicine	[114]
	Amidoxime group, Ti_3_C_2_, grafted polyamide	Excellent anti-biological pollution performance (92.9% antibacterial rate).	Extraction of uranium from seawater	[115]
	Ti_3_C_2_, Al_2_O_3_, Ag, Cu	A total of 99.6% bacteria can be collected in the filter.	Sewage disposal	[116]
	Ti_3_C_2_, tannin	Remarkable antibacterial properties against *E. coli* and *S. aureus*.	Intelligent wearable device	[117]
Chemical synthesis	BiOI, CeO_2,_ Ti_3_C_2_	The photocatalytic bacteriostatic efficiency of *E. coli* and *S. aureus* were 99.76% and 99.89% respectively.	Double static electricity and antifouling of seawater	[118]
	Ti_3_C_2_, Al_2_O_3_, Ag, SiO_2_, Pd	Antibacterial properties.	Ecotoxicology	[119]
	Ag, Ti_3_C_2_, Cu_2_O	Excellent antibacterial activity against *P. aeruginosa* and *S.aureus*.	Antifouling	[120]
	TiO_2_, W_18_O_49_Z, Ti_3_C_2_	The photocatalytic sterilization rate of *E. coil* under illumination is 93.7% (λ ≥ 365 nm, 6 h).	Teleportation	[121]
Layer-by-layer self-assembly	Ti_3_C_2_, dimethyl siloxane	A strong antibacterial effect on *S. aureus* and *E. coli* (≥99% adhered bacterial cells).	Medical implantation	[122]
Electrostatic assembly	Graphite carbonitride, Ti_3_C_2_	In complex water matrix, 7 × 10^7^ CFU/mL of *S. aureus* can be completely inactivated.	Natural water disinfection	[123]
	Fe_2_O_3_, Ti_3_C_2_, glucose oxidase	Simultaneously promote Fe^2+^/Fe^3+^ to penetrate into bacteria and causes planktonic bacteria to die.	Fight stubborn infection	[124]
3D printing	PDA, Ti_3_C_2_, CFPEEK	Effectively kill bacteria after 10 min of NIR irradiation at 808 nm. The antibacterial rate reached 100%.	Orthopedic implant	[125]

### 3.1. Solution Mixing Method

**Figure 7:**
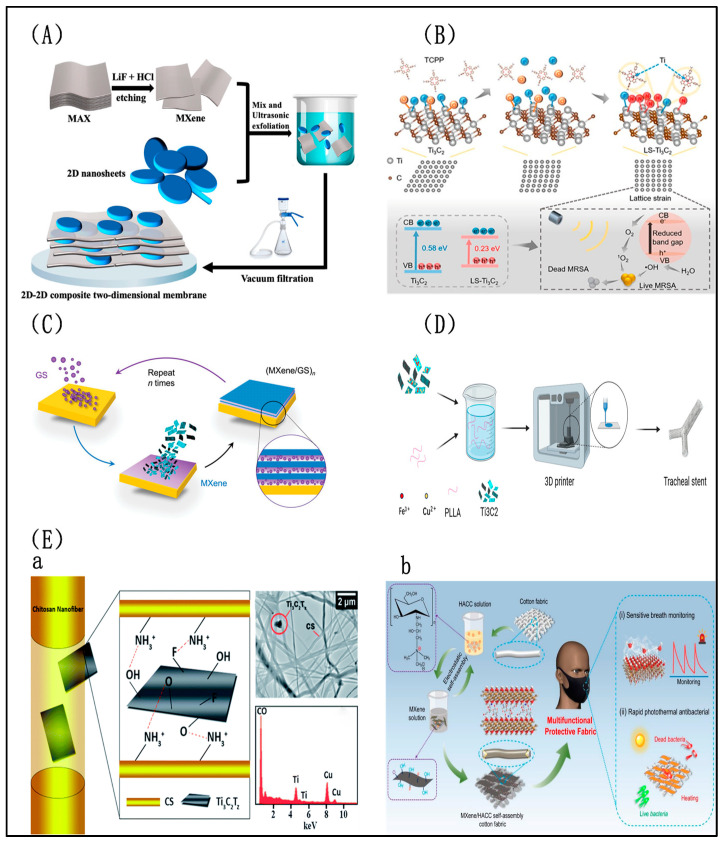
Synthesis methods of Ti_3_C_2_T_x_ composite material [73,122,130,132,141,146]. (**A**) Solution mixing method. (**B**) Chemical synthesis method. (**C**) Layer-by-layer self-assembly. (**D**) Three-dimensional printing method. (**E**) (**a**) Electrostatic spinning method. (**b**) Electrostatic self-assembly.

In this approach, Ti_3_C_2_T_x_ is dispersed in a solution containing other antibacterial agents or materials. Through mixing and subsequent processing, a homogeneous composite material is obtained. This method allows for precise control over the composition and structure of the resulting composite, thereby enabling tailored antibacterial properties. With its advantages of simplicity, low cost, convenience, stability of solution properties, and controllability, the solution mixing method holds great promise for antibacterial applications. Through the solution mixing method, the most biocompatible Ti_3_C_2_T_x_/metal composites can be selected by adjusting the mass ratio of the precursor [50]. Metal nanoclusters integrated with Ti_3_C_2_T_x_ via the solution mixing method induce bacterial membrane damage, instigating the generation of high concentrations of localized ROS. This process effectively oxidizes bacterial membrane lipids, amplifies membrane rupture, and induces severe bacterial DNA fragmentation, ultimately culminating in the demise of Gram-positive and Gram-negative bacteria through a synergistic physical and chemical antibacterial mechanism [126]. Utilizing the solution mixing method, metal oxide CaO_2_ nanoparticles can also be effectively loaded onto the surface of amino-functionalized Ti_3_C_2_T_x_, yielding Ti_3_C_2_T_x_ composite nanosheets endowed with sonochemical/chemical dynamic capability. When subjected to ultrasound treatment for just one hour, these nanosheets prompt the shrinkage, rupture, or complete dissolution of *E. coli* and *S. aureus* [127]. This effect arises from the generation of acoustic sensitizer TiO_2_ on a Ti_3_C_2_T_x_ oxidated surface facilitated by CaO_2_. This, in turn, promotes the Fenton reaction initiated by self-supplied H_2_O_2_, thereby augmenting the production of ROS. Furthermore, the solution mixing method can hybridize two-dimensional Ti_3_C_2_ sheets into a silane film on an aluminum alloy surface. This hybridization exhibits excellent adhesion to the substrate via Si-O-Al chemical bonds, significantly enhancing the thickness and compactness of the silane film, thereby inhibiting both anode and cathode corrosions. Meanwhile, Ti_3_C_2_T_x_ within the film demonstrates excellent antibacterial properties, as evidenced by PI staining, revealing that 90% of *E. coli* were dyed red after 24 h of incubation [128]. In addition, incorporating different mass ratios of Ti_3_C_2_T_x_ nanosheets into epoxy resin through solution mixing and direct curing results in Ti_3_C_2_T_x_/resin composite materials. These materials not only exhibit improved mechanical and abrasive properties compared to pure resin but also manifest increased antibacterial activity with Ti_3_C_2_T_x_ loading, potentially reducing secondary caries caused by bacterial accumulation at the margins of resin composite materials [129]. In the same way, the construction of composite membranes by solution mixing Ti_3_C_2_T_x_ with other two-dimensional materials enhances the permeability and selectivity of substrate membranes, further intensifying antibacterial activity against *E. coli* and *S. aureus* [130] (Figure 7).

### 3.2. Chemical Synthesis Method

Chemical synthesis methods involve blending Ti_3_C_2_T_x_ nanosheets or particles with other materials through chemical reactions to form composite materials. The abundant hydrophilic functional groups on the surface of Ti_3_C_2_T_x_ facilitate tight connections with various semiconductors and strong interaction with water molecules. Common chemical synthesis methods include solvent thermal synthesis and in situ synthesis. Leveraging the controllability, precision, and flexibility of this method enables composite materials with enhanced stability and catalytic performance to be synthesized. For instance, employing the simple wet chemistry method to grow Ag_2_S nanoparticles in situ on the surface of two-dimensional Ti_3_C_2_T_x_ leads to a decrease in the bandgap of the sample with an increase in the Ti_3_C_2_T_x_ content, which subsequently results in a redshift in light absorption and increased light absorption. With the combined action of Ti_3_C_2_T_x_/Ag_2_S and 808 nm NIR light irradiation, the surfaces of *S. aureus* exhibit roughness and varying degrees of pits and damage. Thus, the synergistic antibacterial rate reaches as high as 99.99% [131]. Lattice-strain-rich Ti_3_C_2_T_x_ synthesized utilizing the solvent thermal method greatly enhances the sonochemical effect, thereby realizing extraordinary sonodynamic therapy. The intervention of meso-tetra(4-carboxyphenyl) porphyrin disrupts all Ti-O and most Ti-F chemical bonds on the surface of Ti_3_C_2_T_x_. Recombination between these exposed Ti atoms with amino groups perturbs the order of Ti atoms and leads to Ti atom displacement, ultimately resulting in Ti_3_C_2_T_x_ lattice distortion. This inherent lattice strain narrows the band gap of Ti_3_C_2_T_x_, facilitating the separation and transfer of electron–hole pairs, the generation of ultrasound-mediated ROS, and subsequent robust antibacterial ability against MRSA (99.77 ± 0.16%) [132] (Figure 7). Moreover, a novel Ti_3_C_2_T_x_-based Schottky junction prepared via the solvent thermal method effectively reduces its band gap energy and enhances the photocatalytic activity of Ti_3_C_2_T_x_, achieving inhibitory rates greater than 95% against *E. coli* and *S. aureus* under visible light irradiation [86]. Additionally, a ternary composite material prepared by covalent connection and Schif base reaction exhibits good antibacterial properties against *S. aureus* and *P. aeruginosa*. The covalent bonding between Ti_3_C_2_T_x_ and COFs greatly enhances stability, while the electron transfer channel formed between the ternary materials significantly improves the efficiency of carrier separation, prolonging the lifetime of photogenerated carriers. Furthermore, the slow release of Ag^+^ from a ternary material through electrostatic action maintains long-term antibacterial properties [60].

### 3.3. Layer-by-Layer Self-Assembly Method

Layer-by-layer (LBL) self-assembly stands as a technique employed to fabricate multilayer structures at both nanoscale and microscale levels. Typically, it entails the alternate deposition of Ti_3_C_2_T_x_ and other diverse materials onto a substrate, thereby yielding multilayer films with tailored properties. For example, multilayered crumpled Ti_3_C_2_T_x_ coatings with sharp-edged peaks via LBL self-assembly exhibit on-demand dynamic deformation, and they are capable of removing over 99% of adherent bacterial cells and creating a self-cleaning surface with restored functionality [122] (Figure 7). A multilayer Ti_3_C_2_T_x_/chitosan/Ag coating formed via the self-assembly approach demonstrated outstanding antimicrobial properties, effectively reducing both Gram-negative bacteria (*P. aeruginosa*) and Gram-positive bacteria (*S. aureus*) by 99.9% [133]. Additionally, a poly(L-lactic acid) membrane assembled with positively charged chitosan and negatively charged Ag@Ti_3_C_2_T_x_ exhibited a synergistic antimicrobial effect, resulting in growth inhibition rates of 91.27% against *E. coli* and 96.11% against *S. aureus* [134]. A sandwich-structured superhydrophobic composite film created using superhydrophobic Ti_3_C_2_T_x_ and nanocellulose as raw materials via the LBL self-assembly method demonstrated excellent self-cleaning ability, water repellency, and durability, as well as high photothermal conversion capability and stability. This composite film successfully achieves controllable photo-driven motion and enhanced antimicrobial performance through the simultaneous integration of superhydrophobicity, anti-adhesion, and sustained photothermal sterilization properties [135].

### 3.4. Electrostatic Assembly Method

Electrostatic assembly techniques, notably electrospinning and electrostatic self-assembly, have emerged as pivotal methods for fabricating Ti_3_C_2_T_x_ antibacterial composite materials. Electrospinning technology facilitates the uniform dispersion of Ti_3_C_2_T_x_ nanofibers in a polymer matrix, yielding composite materials with an elevated surface area and robust mechanical properties. Immobilizing Ti_3_C_2_T_x_ on electrospun polycaprolactone membranes [106] or the formation of hybrid Ti_3_C_2_T_x_-based biointerfaces [136,137] makes infected wound microenvironments undergo a transformative shift towards regenerative states, characterized by bacterial eradication, hemostasis, enhanced epithelialization, collagen deposition, and angiogenesis upon NIR light irradiation. Coating Ti_3_C_2_T_x_/Ag [138] or self-assembling Ag@Ti_3_C_2_T_x_ hybrids [139] on other electrospun scaffolds also manifests significant antibacterial effects. Notably, functionalized electrospun nanofiber membranes exhibit high photothermal effects, omnidirectional light harvesting, rapid evaporation rates, and strong antibacterial properties, achieving a notable antibacterial efficiency of 99.9% against *E. coli* after 24 h of contact [140]. Furthermore, wound dressings incorporating delaminated Ti_3_C_2_T_x_ flakes within chitosan nanofibers demonstrate substantial reductions in colony forming units of both Gram-negative *E. coli* and Gram-positive *S. aureus* [141] (Figure 7). Ti_3_C_2_T_x_/zeolite imidazole framework-8 (ZIF-8)/polylactic acid composite membranes exhibit superior PTT and PDT performance, with antibacterial rates of 99.9% and 99.8% against *E. coli* and MRSA, respectively, offering a promising avenue for combating infections by extremely drug-resistant bacteria [142]. Moreover, Ti_3_C_2_T_x_-based electrospun fiber membranes promote the repair and regeneration of peripheral nerve injuries by restoring electrical signal transmission and regulating neuronal membrane function. The addition of Ti_3_C_2_T_x_ enhances the hydrophilicity, conductivity, and biocompatibility of membranes, thereby facilitating cell growth and exhibiting commendable antibacterial properties (as high as 80%) [143]. Piezoelectric stimulation under external ultrasonication augments the growth and proliferation of Schwann cells cultured on these electrospun scaffolds, fostering axonal elongation and myelin sheath formation and ultimately restoring motor and sensory functions in regenerated nerve rats [144].

The electrostatic self-assembly technique involves the sequential deposition of negatively charged Ti_3_C_2_T_x_ and positively charged functional materials, typically chitosan [145] and its derivatives [146] (Figure 7), through electrostatic interactions to form uniform and stable multilayered ultra-thin composite materials. This self-assembly strategy enhances the dispersion of Ti_3_C_2_T_x_ nanosheets, establishing a highly interconnected 3D network that facilitates rapid electron transport pathways. The incorporation of chitosan and Ti_3_C_2_T_x_ confers multifunctionality to the material, including excellent conductivity, sensitivity, mechanical strength (up to 1900%), and flexibility [147]. Except for chitosan, negatively charged Ti_3_C_2_T_x_ can form multifunctional nanomaterials with positively charged metal ions for photothermal antibacterial applications [56]. For example, the integration of Cu^2+^ and Ti_3_C_2_T_x_ into hyaluronic acid hydrogels yields antibacterial dressings with rapid adhesion, self-healing, and inject ability, which can conveniently be applied to wounds of various shapes, providing long-term wound protection. In these antibacterial dressings, the photothermal complexes act as antibacterial barriers, ROS scavengers, and vascular regeneration promoters to accelerate the healing process of infected wounds, ameliorate inflammation, enhance collagen deposition, foster vascular formation, and ultimately facilitate wound closure [59]. Additionally, antibacterial textiles with the adsorption of cationic Bi^3+^ onto Ti_3_C_2_T_x_ nanosheet surfaces exhibit antibacterial efficacy rates of 98.64% and 99.89% against *S. aureus* and *E. coli*, respectively, offering a novel approach for designing light-excited antibacterial textiles [148].

### 3.5. Three-Dimensional Printing

Three-dimensional printing, as a transformative technology for the on-demand fabrication of three-dimensional objects, offers unparalleled advantages in manufacturing complex-shaped and structured items by translating digital designs into tangible objects through layer-by-layer stacking. The integration of Ti_3_C_2_T_x_ with other materials using 3D printing technology has been widely applied in bone tissue engineering [149]. Incorporating Ti_3_C_2_T_x_ into 3D scaffolds enhances the mechanical properties, antibacterial performance, and osteogenicity, showing significant potential for repairing infected bone defects [150]. Multifunctional scaffolds with a sandwich-like structure of Cu_2_O@Ti_3_C_2_T_x_/α-tricalcium phosphate are directly fabricated through 3D printing to achieve programmable temperature control. These scaffolds support brief PTT to swiftly eliminate early bacteria and periodic low-level photothermal stimulation to stimulate bone tissue growth, thereby minimizing damage to healthy cells and tissues. Meanwhile, endogenous copper ions are gradually released inside the scaffolds within a safe dosage range, enhancing the antibacterial effects of early PTT and promoting angiogenesis, ultimately improving the repair outcomes [71]. Personalized Ti_3_C_2_T_x_ composite 3D bio-printed hydrogels with rat bone marrow mesenchymal stem cells have both photothermal antibacterial and osteogenic capabilities to accelerate the healing and bone regeneration of rat mandibular defects infected with *S. aureus* [151]. Poly (lactic acid) tracheal stents uniformly incorporated with CuFe_2_O_4_-Ti_3_C_2_T_x_ heterojunction via selective laser technology deplete glutathione in bacteria by generating high heat and exogenous ROS under NIR light exposure. Furthermore, ROS are continuously produced through a Fenton-like reaction, achieving a robust and persistent antibacterial cascade effect. After only 15 min of NIR light exposure, 96.49% of *S. aureus* and 95.33% of *P. aeruginosa* are eradicated. These heterojunction tracheal stents offer potential prospects for patients with tracheal injuries with the aid of CuFe_2_O_4_-Ti_3_C_2_T_x_ heterojunction [73] (Figure 7). Three-dimensional printing technology has opened up new directions in material design, particularly offering significant advantages in the fabrication of items with complex shapes and structures, and it holds limitless potential for various applications.

At present, these are the only common synthesis methods for Ti_3_C_2_T_x_ composite antibacterial materials (Figure 7), but it is believed that with the development of science and continuous in-depth research by researchers, more synthesis methods will be found to cope with more scenarios and broaden the application prospect of Ti_3_C_2_T_x_ composite antibacterial materials.

The preparation methods for Ti_3_C_2_T_x_-based composite antibacterial materials have evolved from simple mixing to chemical reactions, self-assembly, and finally, customization through 3D printing. In particular, 3D printing technology enables the direct printing of Ti_3_C_2_T_x_ with polymers into composite structures, achieving precise control over the structure and performance of antibacterial materials, providing greater flexibility and customization for their applications, and offering insights and inspiration for the design and preparation of future antibacterial materials.

## 4. Application of Ti_3_C_2_T_x_ Composite in Antibacterial Field

**Figure 8:**
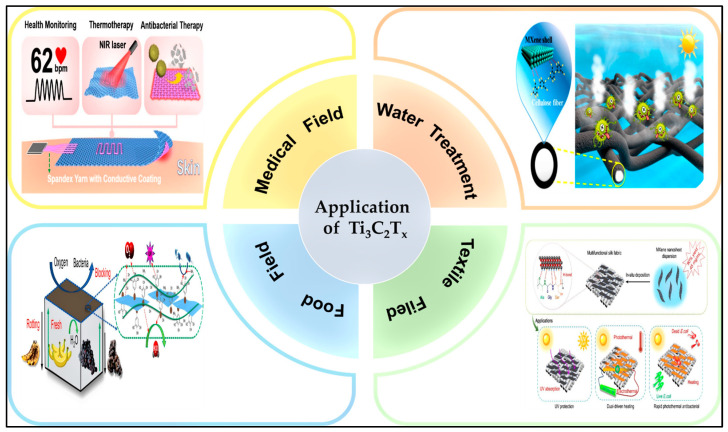
Application of Ti_3_C_2_T_x_ composite antibacterial materials [46,152,153,154].

With its unique physical structure and chemical characteristics, Ti_3_C_2_T_x_ has a broad application prospect in the antibacterial field, including in the medicine, water treatment, textile, and food industries and other aspects (Figure 8).

### 4.1. Medical Field

With the continuous progress of medicine, there are more and more requirements for antibacterial properties. As a new nanomaterial, Ti_3_C_2_T_x_ shows great potential in the field of medical antibacterial materials.

#### 4.1.1. Wound Healing

Ti_3_C_2_T_x_ exhibits the ability to prevent bacterial resistance by inducing bacterial death through mechanical damage and oxidative stress. Moreover, Ti_3_C_2_T_x_, which is rich in functional groups, has great potential for modification with molecules such as proteins, growth factors, and nanoparticles. These properties have attracted more and more attention from researchers, who are ready to apply them in the modification and construction of wound dressings against multidrug-resistant bacteria [155]. For example, immobilizing lysozyme on the hybrid interface of Ti_3_C_2_T_x_ has been shown to enable the precise control of local heat and enhance the photothermal response of loaded lysozyme biocatalysis, effectively inhibiting the proliferation of MRSA and accelerating the disinfection of mouse wounds [156]. This enhancement is mainly attributed to the photothermally enhanced lysozyme activity, along with the bacterial death caused by local mild hyperthermia and physical destruction. The excellent photothermal effect of Ti_3_C_2_T_x_ provides new design and application ideas for skin wound dressing. During wound healing, Ti_3_C_2_T_x_ increases the material temperature to 40 °C or higher, significantly inhibiting bacterial growth activity. A multifunctional polyvinylidene fluoride (PVDF) membrane loaded with chitosan–Ti_3_C_2_T_x_ suspension exhibits an inhibitory ability close to 100% under NIR irradiation. Animal experiment results demonstrate that the wound healing rate reaches 95% with the aid of NIR irradiation for 14 days, which is markedly higher than that of the group without NIR irradiation [145]. Moreover, the surface charges of Ti_3_C_2_T_x_ promote blood cell aggregation, platelet activation, and clot formation. The novel composite membrane containing graphene oxide and Ti_3_C_2_T_x_ significantly increases tensile strength, platelet adsorption, and the blood clotting time in addition to having a 97% antibacterial rate against both Gram-positive and Gram-negative bacteria [157]. Additionally, multifunctional hydrogels based on regenerated bacterial cellulose and Ti_3_C_2_T_x_ have good mechanical properties, flexibility, biodegradability, and high water absorption capacity. These hydrogels promote skin wound healing through the electrical stimulation of skin cell behavior with the help of the electrical conductivity of Ti_3_C_2_T_x_. These hydrogels significantly enhance the proliferation activity of NIH3T3 cells and accelerate the wound healing process when coupled with electrical stimulation, providing an effective synergistic treatment strategy and proving that they are attractive candidates for wound dressings [93]. Furthermore, since abundant functional groups on the surface of Ti_3_C_2_T_x_ can react with ROS, the addition of Ti_3_C_2_T_x_ to skin wound dressings can improve the overall ROS scavenging ability of the dressings [158]. In summary, Ti_3_C_2_T_x_-based composite antimicrobial materials, with their excellent biocompatibility, photothermal effect, and electrical conductivity, effectively reduce the risks of bacterial adhesion and infection, improving wound healing and patient recovery times.

#### 4.1.2. Medical Sensor

Ti_3_C_2_T_x_ composite antibacterial materials offer significant advantages in the development of electrochemiluminescence sensors [159], flexible electronic devices [160], and wearable skin sensors [161] due to their outstanding electrochemical and antibacterial properties. Sensors constructed from Ti_3_C_2_T_x_ composites have the capability to monitor human movements in real time, encompassing large deformations (e.g., finger, elbow, wrist, and knee bending) and small deformations (such as mouth movements and throat sounds) [52]. These sensors are applied in real-time movement detection in amyotrophic lateral sclerosis patients, writing encryption, and human–machine information transfer [162]. Electrospun nanofiber strain sensors with a Ti_3_C_2_T_x_-based conductive interlayer demonstrate remarkable performance in gesture language recognition [163]. Moreover, multifunctional, robust, flexible knitted strain sensors composed of conductive Ti_3_C_2_T_x_ nanosheets serve as intelligent sensing gloves for accessible communication among individuals with impaired hearing [164]. Conductive organic hydrogels based on Ti_3_C_2_T_x_ nanosheets exhibit the highest pressure-sensing performance (S-p1 = 782.7 kPa^−1^) and ultra-low-temperature tolerance (−81 °C). Zinc-activated Ti_3_C_2_T_x_ nanosheets endow hydrogel sensors with good antibacterial capabilities and have potential to be used for medical applications [165]. Radiation-synthesized poly(ionic liquid)/Ti_3_C_2_T_x_ gels display excellent antifreeze properties (−60 °C), durability (90 days at room temperature), and outstanding antibacterial activity against *E. coli* (99.82%) and *S. aureus* (99.98%). Multifunctional sensors based on these hybrid gels offer real-time and rapid response capabilities in detecting various human activities, recognizing writing strokes, and facilitating encrypted information transmission [166]. Capacitive pressure sensors prepared using carbon nanotubes, Ti_3_C_2_T_x_, and polydimethylsiloxane composites feature an ultra-low detection limit, a wide detection range, high sensitivity, a fast response time, and excellent stability. These sensors accurately recognize mechanical stimuli while meeting the requirements for elasticity, antibacterial activity, wearing comfort, and long-term stability, thus providing innovative solutions for monitoring human activities and detecting diseases non-invasively [167]. Some sensors also possess superhydrophobic and anti-fouling properties, enabling them to resist interference from water droplets and flow [168], thereby making them suitable for use in humid or rainy conditions and complex environments [169].

Ti_3_C_2_T_x_ composite-based sensors also demonstrate impressive photothermal conversion capabilities. Under NIR irradiation (808 nm, 0.2 W/cm^2^) for 10 s, they reach a temperature of 55 °C, effectively achieving the complete sterilization of both *S. aureus* and *E. coli* [170]. These sensors hold potential for further development into a diagnostic and treatment system that monitors human movement while simultaneously providing thermotherapy and antibacterial treatment at the site of motion injury [171]. The introduction of noble metal Ag nanoparticles, leveraging the synergistic photothermal effects of Ti_3_C_2_T_x_ and Ag, enhances photothermal energy storage and antibacterial properties, making this method suitable for moderate PTT and pain relief [172]. The exceptional electrical conductivity and distinct plasma effects of metal nanoparticles have amplified sensor sensitivity by fourfold. Within the range of 0–100 kPa, the linear sensitivity measures 24.5 kPa^−1^. Additionally, there is a 25.3 °C electrothermal enhancement at 7.5 V and a 5.6 °C photothermal enhancement at 100 mW cm^−2^. Moreover, these enhancements result in an antibacterial efficiency of up to 99.9% [36]. Strain sensors assembled with Ti_3_C_2_T_x_ nanosheets and PDA/Ni^2+^ demonstrate strong antibacterial effects, rapid response to NIR radiation, and controllable surface temperatures, showing great potential for on-demand thermotherapy [152] (Figure 8). Epidermal sensors assembled from antibacterial Ti_3_C_2_T_x_ hydrogels can sensitively monitor human movements during rehabilitation training, detect tiny electrophysiological signals for the diagnosis of cardiovascular and muscle-related diseases, facilitate wearable human–machine interaction, and treat wound infections to promote wound healing [173]. Wireless flexible sensors made of Ti_3_C_2_T_x_-based hydrogels, benefiting from the synergistic effect of antibacterial properties and electrical stimulation, provide potent angiogenic effects for diabetic wounds, promote collagen production, and accelerate wound healing, thus effectively safeguarding high-risk populations [174].

#### 4.1.3. Medical Implant Field

Ti_3_C_2_T_x_ composite antibacterial materials hold broad application prospects in the field of medical implants due to their good biocompatibility. A 2019 study first explored the application of Ti_3_C_2_T_x_ films in bone tissue engineering and guided bone regeneration therapy. This study confirmed the high cell compatibility of Ti_3_C_2_T_x_ films and their capacity to foster osteogenic differentiation in vitro. After the implantation of Ti_3_C_2_T_x_ membranes into rat subcutaneous and cranial defect sites, they exhibited favorable biocompatibility, osteoinductivity, and bone regeneration activity in vivo [175]. Infections present a substantial risk to individuals with medical implants, potentially leading to implant failure, tissue necrosis, and even amputation. Ti_3_C_2_T_x_ can mitigate and manage microbial biofilms on indwelling implantable materials and devices by its exceptional antibacterial properties, effectively addressing both antibacterial and bone integration [176]. Crumpled Ti_3_C_2_T_x_ coatings on implants exert robust antibacterial effects against *S. aureus* and *E. coli*, effectively eliminating >= 99% of adherent bacterial cells and restoring surface functionality [122]. Various thicknesses of few-layer Ti_3_C_2_T_x_ coatings, fabricated through anodic electrophoretic deposition, notably enhance the initial adhesion and proliferation capabilities of osteogenic precursor cells such as MC3T3-E1. Moreover, they effectively inhibit the adhesion and cell viability of *S. aureus* and drug-resistant strains, with the antibacterial efficacy directly correlating with the Ti_3_C_2_T_x_ content [177]. Integrating Ti_3_C_2_T_x_ nanosheets with other materials to formulate composite coatings represents a promising strategy for antibiotic-free antibacterial treatment in orthopedic implants, effectively combating pathogenic infections and circumventing antibiotic resistance. For example, embedding plasma-coated cobalt nanowires with Ti_3_C_2_T_x_ can rapidly capture photogenerated electrons from Ti_3_C_2_T_x_ nanosheets, inhibit hot electron–hole recombination, and promote charge carrier transfer under 808 nm NIR irradiation, thereby increasing the yield of bactericidal ROS [178]. CoFe_2_O_4_/Ti_3_C_2_T_x_ nanosheet-modified hydrogels spin-coated onto hard tissue implant materials endow the implants with potent anti-pathogen capabilities through PTT local heat, PDT, and chemodynamic therapy ROS accumulation properties [179]. Ti_3_C_2_T_x_ composite hydrogel scaffolds loaded with rat bone marrow mesenchymal stem cells using 3D printing technology demonstrate a reliance on the biocompatibility and osteogenic capacity of Ti_3_C_2_T_x_. This synergy of antibacterial and osteogenic activities in vivo promotes the healing and bone regeneration of rat mandibular defect infections with *S. aureus* [151]. Beyond bone implants, Ti_3_C_2_T_x_ demonstrates promising potential for neural repair in neural catheter implant applications, attributed to its outstanding conductivity. Composite fiber membranes, incorporating varying Ti_3_C_2_T_x_ contents via electrospinning, contribute to the repair and regeneration of peripheral nerve injuries by restoring electrical signal transmission and regulating neuronal membrane functions. The addition of Ti_3_C_2_T_x_ improves the hydrophilicity and conductivity of electrospun composite membranes, fostering favorable biocompatibility for cell growth while achieving an antibacterial rate of up to 80%, effectively preventing post-implantation wound inflammation [143].

### 4.2. Water Treatment

As a novel two-dimensional nanomaterial, Ti_3_C_2_T_x_ has superior performance in water treatment, particularly when prepared with Ti_3_C_2_T_x_ antibacterial composites. Currently, Ti_3_C_2_T_x_ antibacterial composite materials are primarily utilized in seawater desalination and sewage treatment.

#### 4.2.1. Seawater Desalination

Water scarcity has become a global urgent concern, exacerbated by the relentless progression of society and economy, which intensifies the imbalance between water supply and demand. Against this backdrop, seawater desalination technology has emerged as a pivotal solution to mitigate water scarcity. Sustainable solar-driven seawater desalination technology stands out as a promising avenue to overcome freshwater resource shortages [180]. Nonetheless, solar water evaporation materials encounter serious challenges, such as scaling, low freshwater yield, and inadequate long-term performance in seawater [181]. Ti_3_C_2_T_x_ composites boast high photothermal conversion efficiency, substantially reducing energy consumption in the seawater desalination process, thereby heralding a technological revolution in solar thermal desalination [182]. The Ag/Ti_3_C_2_T_x_/aromatic nanofiber composite aerogel photothermal evaporator, characterized by internal 3D micropores and layered structures, showcases exceptional and efficient salt resistance, desalination, antibacterial properties, and heavy metal ion wastewater purification capabilities. Its excellent thermal stability ensures resilience across a wide temperature range from −500 °C to 196 °C [183]. Inspired by natural trees, a biomimetic solar-driven interfacial evaporator has been successfully developed using vertically aligned hydrophilic sodium alginate aerogel and a layered assembly of Ti_3_C_2_T_x_-interwoven carbon nanotube networks as a micro-nano photothermal absorption layer. This innovative design exhibits excellent water evaporation performance, reaching up to 2.416 kg m^−2^ h^−1^ (after subtracting dark conditions) with a solar energy utilization efficiency of up to 90.56%, which is far higher than that of most reported seawater desalination devices. Furthermore, the solar-driven evaporator boasts robust antibacterial properties and long-term operational stability [184]. An array of Janus photothermal conversion materials with asymmetric characteristics has gradually attracted the interest of researchers. Notably, the Janus photothermal porous fiber membrane, composed of a hydrophobic Ti_3_C_2_T_x_/polydimethylsiloxane coating and a hydrophilic polylactic acid/TiO_2_ nanofluid porous fiber membrane, exhibits a stable photothermal conversion efficiency of 60% with a freshwater yield of 1 kg m^−2^ h^−1^ and an ion concentration of <=1 ppm under direct one-sun irradiation, outperforming other membranes reported in the literature. With the synergistic effect of TiO_2_ nanofluid and Ti_3_C_2_T_x_, the Janus fiber membrane achieves a salt rejection rate of up to 99.95% and antibacterial activity of up to 100% [185]. Moreover, the waste biomass solar evaporator with a Janus structure made from porous pomelo peel and a Ti_3_C_2_T_x_ coating has an evaporation rate of 1.48 kg m^−2^ h^−1^ and a photothermal conversion efficiency of 92.3%. Even after 50 cycles under simulated one-sun lighting, it sustains high evaporation performance and exhibits excellent antibacterial properties [186].

#### 4.2.2. Sewage Disposal

As the contamination of the environment by industrial pollutants and organic dyes continues to escalate, the desire for clean water resources becomes increasingly urgent. In recent years, photocatalysis has emerged as a predominant method for addressing water pollution, with Ti_3_C_2_T_x_ standing out due to its dual functionality in photocatalytic degradation and antibacterial action [187]. Ti_3_C_2_T_x_ exhibits significant advantages in wastewater treatment, effectively eliminating organic pollutants, heavy metal ions, and bacteria from contaminated water sources [105]. Micron-thick Ti_3_C_2_T_x_ membranes, filtered on a PVDF carrier, enhance the antibacterial activity of the PVDF membrane against *Bacillus subtilis* (*B. subtilis*) and *E. coli* by more than 73% and 67%, respectively. This underscores the potential of Ti_3_C_2_T_x_-modified membranes not only in combating common waterborne bacteria but also as antifouling membranes in wastewater treatment processes [188]. Furthermore, the combination of Ti_3_C_2_T_x_ with cellulose to form Ti_3_C_2_T_x_/cellulose photothermal membranes achieves nearly 94% light absorption efficiency and demonstrates antibacterial efficiency exceeding 99%, bringing promising prospects for practical application in long-term wastewater treatment [153] (Figure 8). Composite membranes formed by combining Ti_3_C_2_T_x_ nanosheets with silver nanowires [189] or nanoparticles [190] exhibit an inhibition rate exceeding 99% against *E. coli* and *S. aureus*, positioning them as attractive candidates for developing nanofiltration membranes for water purification and biomedical applications. Moreover, Ti_3_C_2_T_x_-functionalized phages, prepared by loading Ti_3_C_2_T_x_ nanoparticle fragments onto well-characterized phages via electrostatic bonding, have high specificity for the recognition of host receptors, the targeting of phage and bacterial surface negative charges [191]. This approach can significantly increase the adsorption rate and stability of phages in aquatic environments, demonstrating robust antibacterial effects on bacterial cell targets and reducing artificial contamination in water samples by 99.99%. To scale up wastewater treatment efforts, researchers have designed a simple yet effective antibacterial nanorobot by loading ultra-small gold nanorods onto the surface of Ti_3_C_2_T_x_ nanosheets. This nanorobot enhances light absorption cross-sections, improving mobility and antibacterial capabilities under irradiation. Compared to traditional static antibacterial methods, this unique self-propelled capability offers an effective and sustainable approach for treating bacterial pollution in water on a large scale [192].

### 4.3. Textile Field

The pressing demand for multifunctional and high-performance wearable heaters in future human health applications is undeniable. Wearable heaters, fashioned by decorating fibers with Ti_3_C_2_T_x_ through a straightforward solution dipping coating technique, hold promising potential and stand as an optimal choice for forthcoming health management and safeguarding endeavors. The Ti_3_C_2_T_x_-decorated fabrics boast commendable dual-driven energy conversion capabilities, seamlessly integrating photothermal and electrothermal functionalities, which achieve an impressive antibacterial efficiency surpassing 99% against Gram-negative *E. coli* within a mere 20 min of contact [46] (Figure 8). These fabrics also excel in electromagnetic interference shielding, demonstrating an efficiency of 42.1 dB in the x-band [193]. A triboelectric nanogenerator, utilizing fabric as the electrode, converts open-circuit voltage into an electrical signal by harnessing the energy from humans’ tiny movements, showcasing immense potential for self-powered electronic device applications [194]. Customized Janus textiles with asymmetric optical properties precisely regulate body temperature to adapt seamlessly to fluctuating climatic conditions, rendering them indispensable for optimal personal thermal management and climate resilience efforts [195]. Furthermore, self-disinfecting textiles, harnessing synergistic photodynamic and photothermal effects, herald a breakthrough in healthcare applications. Antibacterial medical textiles, achieved through the curling of exfoliated 2D Ti_3_C_2_T_x_ MXene sheets on polypropylene fibers, exhibit unparalleled efficacy in reducing bacterial viability by 100% through physical contact and light-induced ROS generation [196]. Intelligent photochromic self-disinfecting textiles, engineered through the in-situ growth of MOF, electro-sprayed Ti_3_C_2_T_x_ colloid, and screen-printed thermochromic dyes, demonstrate the capability to neutralize 99.9999% of Gram-negative (*E. coli* ATCC 8099) and Gram-positive (*S. aureus* ATCC 6538) bacteria within 30 min [87]. Mechanism insights reveal that light-driven singlet oxygen and thermal effects are pivotal in bacterial inactivation. Combining Ti_3_C_2_T_x_ quantum dots with potent antibacterials forms an organic–inorganic hybrid layer on fiber materials. This enables textiles to exhibit varying fluorescence recovery capabilities following exposure to different pathogen concentrations, achieving a remarkable bactericidal efficiency exceeding 99.99% against both *E. coli* and *S. aureus* within a brief time frame of 30 min [197]. Additionally, pioneering the exploration of multifunctional coatings via composite nanomaterial design holds the key to advancing the next generation of polyester textiles. Polyester textiles, assembled with MoO_3_-x quantum dots on Ti_3_C_2_T_x_, exhibit exceptional bactericidal efficacy exceeding 99% against *E. coli* and *S. aureus* under sunlight, while concurrently mitigating smoke by 36.16% [198]. Nonwoven fabric created by combining a conductive material such as Ti_3_C_2_T_x_ with traditional textile PET achieves remarkable antibacterial efficiency surpassing 99.99% against *E. coli* under simulated sunlight exposure at a rate of 300 mW/cm^2^. The formation of a dense protective Ti_3_C_2_T_x_ layer during combustion significantly enhances fabric flame retardancy, showcasing boundless potential in crafting adaptive wearable technologies [199].

### 4.4. Food Filed

The globalization of markets and the surge in global population have spurred an escalating demand for food, presenting significant challenges in ensuring food safety. Amid this backdrop, averting bacterial contamination emerges as a prevalent strategy to prolong the shelf life of food items and uphold their quality. Integrating Ti_3_C_2_T_x_ particles into a polylactic acid polymer matrix to craft a composite film not only maintains the thermal and mechanical properties of polylactic acid but also provides the film with the inherent antibacterial prowess of Ti_3_C_2_T_x_. Remarkably, a mere 0.5 wt.% of Ti_3_C_2_T_x_ is adequate to achieve a staggering 99.9999% bactericidal activity against *Listeria monocytogenes* (a six-log reduction), while for *Salmonella*, only 5 wt.% of Ti_3_C_2_T_x_ suffices to attain a 99.999% bactericidal efficacy (a five-log reduction) [200]. Furthermore, the amalgamation of Ti_3_C_2_T_x_ and tannic acid into chitosan networks via hydrogen bonding and electrostatic self-assembly engenders biopolymer-based composite films with substantially enhanced antibacterial and antioxidant capabilities. These films boast exceptional water vapor and oxygen barrier properties, effectively extending the shelf life of perishable produce like bananas and grapes. Consequently, these composite films emerge as ideal solutions for storing fruits and vegetables [154] (Figure 8). Moreover, Ti_3_C_2_T_x_ exhibits the remarkable ability to impede ice crystal formation and growth, thereby mitigating cryoinjury during cooling and thawing, which could otherwise jeopardize stem cells. This effect translates into a notable enhancement in cell viability, elevating it from 38.4% to 80.9%. Additionally, Ti_3_C_2_T_x_ MXene showcases synergistic antibacterial activity under laser irradiation, facilitating the sterile cryopreservation of stem cells [201].

### 4.5. Other Filed

In the realm of drug delivery, Ti_3_C_2_T_x_ composite antibacterial materials provide a versatile platform for developing drug delivery systems. They enable targeted drug delivery, diminish drug toxicity, and enhance the pharmacokinetics of drug molecules. By grafting doxorubicin (DOX) as a model drug onto functionalized Ti_3_C_2_T_x_ nanosheets, a nanoparticle with exceptional drug loading and encapsulation efficiency, as well as outstanding dual (ROS- and pH-responsive) properties, is achieved. Moreover, Ti_3_C_2_T_x_-DOX nanosheets exhibit excellent antibacterial activity against both Gram-negative *E. coli* and Gram-positive *B. subtilis* [202]. Incorporating Ti_3_C_2_T_x_ into a modified polyurethane enhances its physicochemical properties and biocompatibility. This composite film controls drug release from the polymer matrix during gradual degradation and swelling, facilitating precise drug delivery to the site of the disease, thereby improving therapeutic efficacy and minimizing damage to healthy tissues [203]. Expanding into the industrial sector, the application of Ti_3_C_2_T_x_ materials has broadened, particula_r_ly in fabricating composites with high-performance antibacterial and antioxidant properties. An innovative strategy involves intercalating Ti_3_C_2_T_x_ into montmorillonite layers to form a nanocomposite with a micro-nano protective structure. This composite material exhibits remarkable antibacterial efficacy, with a suppression rate exceeding 99.9% against *S. aureus*. This high antibacterial performance persists for up to 28 days. Additionally, these composites demonstrate potent antioxidant activity, providing a solid theoretical foundation and practical application potential for the preparation and maintenance of valuable furniture [204]. Furthermore, the development of a copper–organic phosphate–Ti_3_C_2_T_x_ heterojunction has ushered in new opportunities for the industrial sector. This heterojunction not only achieves over 99.9% antibacterial efficiency, but also yields epoxy resin nanocomposites with satisfactory flame-retardant properties. Such a material, coupling high antibacterial and flame-retardant performance, creates more opportunities for applications in industries such as coatings, medical devices, and furniture. It enhances product safety, durability, and inspires new ideas and solutions for product innovation in these sectors [205].

Ti_3_C_2_T_x_-based composite antibacterial materials possess broad application prospects, spanning from food preservation to seawater desalination, wastewater treatment, and medical fields such as drug delivery systems, wound dressings, medical sensors, and bone implants, and they even have significant applications in industrial sectors. In the future, Ti_3_C_2_T_x_ composite antibacterial materials are expected to achieve breakthroughs in more frontier fields with their excellent antibacterial properties and wide application potential.

## 5. Conclusions

In summary, Ti_3_C_2_T_x_ materials exhibit tremendous potential in the field of antibacterial applications due to their unique physical exfoliation, photodynamic, and photothermal properties. However, inherent limitations such as inadequate solar response capability and carrier separation efficiency have constrained their photocatalytic performance, leading to suboptimal antibacterial efficacy. To overcome these intrinsic constraints, researchers have significantly enhanced the antibacterial properties of Ti_3_C_2_T_x_ composite materials through compositional diversification and modifications in composite methodologies. Over the past decade, Ti_3_C_2_T_x_ composite materials have made rapid strides in antibacterial applications, not only achieving breakthroughs in material compositional diversity but also demonstrating extensive possibilities in practical applications. As technology matures and scientific research deepens, the research and application prospects of Ti_3_C_2_T_x_ composite antibacterial materials are expected to broaden further, promising significant contributions to human health, environmental protection, and industrial development.

## 6. Challenges and Prospects

Ti_3_C_2_T_x_ composite antibacterial materials, as a promising nanomaterial with broad application potential in the antibacterial field, have made significant breakthroughs. However, several challenges remain. With continuous technological advancements, further enhancing the performance of Ti_3_C_2_T_x_ composite antibacterial materials can promote their widespread application and development in fields such as medicine, environmental protection, and daily essentials as follows:

(1) Structure optimization for customized design: A deep understanding of the relationship between the microstructure and properties of materials enables precise control of the structural parameters of composite materials, such as nano-scale composite components, morphology, and crystal structure, to achieve customized designs of material properties. Utilizing computational simulation, biomimetic design, and modern material characterization techniques, such as transmission electron microscopy and atomic force microscopy, enables the multi-scale characterization, prediction, and optimization of the structures and properties of Ti_3_C_2_T_x_ composite antibacterial materials. This comprehensive understanding of the structure–property relationship facilitates precise control tailored to the requirements of various application scenarios.

(2) The in-depth exploration of antibacterial mechanisms: With the continuous development of biotechnology and materials science, future research will focus on understanding the release mechanisms and control strategies of antibacterial active substances in Ti_3_C_2_T_x_ composite materials at the molecular level, utilizing methods such as computational simulation and molecular dynamics simulation. Effective methods will be sought to precisely control the release rate, amount, and mode of antibacterial active substances. The interaction processes between antibacterial active substances and bacteria, especially drug-resistant bacteria, will be thoroughly explored to reveal the possible adaptability and evolution processes of bacteria to the materials and to study the action mechanisms of the materials on drug-resistant bacteria. Additionally, considering that Ti_3_C_2_T_x_ composite antibacterial materials may possess multiple antibacterial mechanisms, such as physical capture, chemical reaction, photocatalysis, etc., future research will focus on exploring the interactions and synergistic effects among these different antibacterial mechanisms. This will further enhance the antibacterial effectiveness of the materials and expand their applicability in different scenarios.

(3) The synergistic optimization of biocompatibility and antibacterial performance: Currently, Ti_3_C_2_T_x_ composite antibacterial materials are increasingly used in the field of human implants. However, there is limited research on their biocompatibility, especially regarding their long-term usage. In the future, it is necessary to focus on establishing and improving biocompatibility evaluation standards and to explore how surface modification and functionalization can be utilized to control parameters such as nanoscale structure and surface charge, thereby improving the interface properties between the material and the biological system to reduce immune reactions and cell toxicity. Additionally, future research could explore synergistic optimization strategies between biocompatibility and antibacterial performance. This entails modulating the material’s structure, composition, and surface properties to achieve the dual optimization of biocompatibility and antibacterial performance.

(4) The optimization of preparation processes for green and environmentally friendly outcomes: Utilizing green solvents, such as water and ethanol, as replacements for traditional organic solvents reduces environmental pollution and chemical usage. Optimizing process parameters, such as temperature, time, and pressure, can decrease energy consumption and exhaust emissions. Additionally, exploring novel preparation methods like 3D printing and electron beam irradiation techniques enables the precise control and functional modification of the structures of Ti_3_C_2_T_x_ composite materials, enhancing their practicality and specificity. These approaches provide more expedient and efficient solutions and market prospects for the application of materials.

In conclusion, Ti_3_C_2_T_x_ composite antibacterial materials face a series of challenges but also have broad application prospects. Through the continuous optimization of preparation processes, structural and performance regulation, and in-depth research into antibacterial mechanisms, Ti_3_C_2_T_x_ composite antibacterial materials will play a role in more fields, contributing to the development of human society.
